# Isolation of New Gravitropic Mutants under Hypergravity Conditions

**DOI:** 10.3389/fpls.2016.01443

**Published:** 2016-09-29

**Authors:** Akiko Mori, Masatsugu Toyota, Masayoshi Shimada, Mika Mekata, Tetsuya Kurata, Masao Tasaka, Miyo T. Morita

**Affiliations:** ^1^Graduate School of Bioagricultural Sciences, Nagoya UniversityNagoya, Japan; ^2^Department of Botany, University of WisconsinMadison, MadisonWI, USA; ^3^Japan Science and Technology Agency, Precursory Research for Embryonic Science and TechnologySaitama, Japan; ^4^Graduate School of Biological Sciences, Nara Institute of Science and TechnologyIkoma, Japan; ^5^Graduate School of Life Sciences, Tohoku UniversitySendai, Japan; ^6^CREST, Japan Science and Technology AgencyTokyo, Japan

**Keywords:** gravitropism, enhancer mutant, hypergravity, whole genome sequencing, *Arabidopsis thaliana*

## Abstract

Forward genetics is a powerful approach used to link genotypes and phenotypes, and mutant screening/analysis has provided deep insights into many aspects of plant physiology. Gravitropism is a tropistic response in plants, in which hypocotyls and stems sense the direction of gravity and grow upward. Previous studies of gravitropic mutants have suggested that shoot endodermal cells in *Arabidopsis* stems and hypocotyls are capable of sensing gravity (i.e., statocytes). In the present study, we report a new screening system using hypergravity conditions to isolate enhancers of gravitropism mutants, and we also describe a rapid and efficient genome mapping method, using next-generation sequencing (NGS) and single nucleotide polymorphism (SNP)-based markers. Using the *endodermal-amyloplast less 1* (*eal1*) mutant, which exhibits defective development of endodermal cells and gravitropism, we found that hypergravity (10 g) restored the reduced gravity responsiveness in *eal1* hypocotyls and could, therefore, be used to obtain mutants with further reduction in gravitropism in the *eal1* background. Using the new screening system, we successfully isolated six *ene* (*enhancer of eal1*) mutants that exhibited little or no gravitropism under hypergravity conditions, and using NGS and map-based cloning with SNP markers, we narrowed down the potential causative genes, which revealed a new genetic network for shoot gravitropism in *Arabidopsis*.

## Introduction

Plants alter the orientation of their growth in response to environmental stimuli, such as light, moisture, and gravity. Gravitropism is a well-known growth response in which shoots grow upward and roots grow downward, according to the direction of gravity, and the molecular mechanism of gravitropism has been investigated for decades, using physiological and genetic methods in *Arabidopsis thaliana* (Blancaflor and Masson, 2003; Morita, 2010). One method, forward genetics, is a powerful approach used to clarify relationships between phenotypes and genes and has contributed to a number of significant findings in plant physiology, including gravitropism. Null mutants of the GRAS family transcription factor SGR7/SHR, *shoot gravitropism* (*sgr*)*7-1*/*short root* (*shr*)*-2*, lack endodermal cells and fail to exhibit gravitropism in their hypocotyls and stems (Fukaki et al., 1998; Helariutta et al., 2000), and *endodermal-amyloplast less 1* (*eal1*) mutants have a single amino acid deletion in SGR7/SHR that results in abnormal development of the endodermis and insufficient gravitropic responses (Fujihira et al., 2000; Morita et al., 2007). Shoot endodermal cells contain starch-filled plastids (amyloplasts) that distribute toward the gravity vector. The *sgr2* and *zig/sgr4* mutants have immobile amyloplasts in their endodermal cells, owing to abnormal vacuolar dynamics, and exhibit reduced shoot gravitropism (Saito et al., 2005; Toyota et al., 2013b). Amyloplasts in *sgr9* mutants exhibit irregular interactions with actin filaments, which results in non-sedimentable amyloplasts and weak gravitropism (Nakamura et al., 2011). Comprehensive analyses of these gravitropic mutants support the idea that endodermal cells are gravity-sensing cells, i.e., statocytes, in *Arabidopsis* shoots and that the sedimentation of amyloplasts plays a crucial role in gravity perception. However, the main signaling pathway following amyloplast sedimentation remains largely unknown, since conventional screening has not identified such mutants/genes. Therefore, to isolate novel mutants/genes in gravity sensing/signaling, it is necessary to develop a new screening system under different gravitational conditions.

Hypergravity created by centrifugation provides a unique experimental environment that can be used to investigate the gravity responsiveness of plants living on Earth (1 g). Knight (1806) built a centrifuge (waterwheel) that was driven by the flow of a river and used it to determine that plants perceive gravitational and centrifugal acceleration and exhibit gravitropic responses. The starchless mutants, *phosphoglucomutase* (*pgm*), exhibit reduced gravity sensitivity in hypocotyls and roots at 1 g, which is restored by hypergravity, such as 10 g (Fitzelle and Kiss, 2001), and *sgr2* mutants exhibit little gravitropism at 1 g, whereas hypergravity (10 and 30 g) moved the immobile amyloplasts of *sgr2* mutants toward the gravity vector and induced obvious gravitropism in stems (Toyota et al., 2013b). However, *sgr7-1/shr-2* mutants failed to exhibit gravitropism in stems, even at 30 g, which suggests that *sgr7-1/shr-2* mutants completely lack gravity sensing machinery (Toyota et al., 2013b). Interestingly, the *eal1* mutant, a weak allele of *sgr7-1/shr-2*, exhibits reduced but distinguishable gravitropism in hypocotyls at 1 g (Fujihira et al., 2000), which indicates that *eal1* mutants still possess gravity sensing machinery in their abnormal endodermal cells.

In the present study, we designed a new screening system that used hypergravity conditions to search for enhancing mutations that further reduce the weak gravitropism of *eal1* hypocotyls. We found that hypergravity intensified hypocotyl gravitropism in both wild-type and *eal1* plants and that it enabled enhancer screening of the gravitropic mutant *eal1*. In addition, we also utilized whole genome sequencing and ethyl methanesulfonate (EMS)-derived single nucleotide polymorphism (SNP) markers to identify the causative mutations of the enhanced phenotypes (Hartwig et al., 2012). We report that six *ENHANCER OF EAL1* (*ENE*) genes are related to gravitropism, two of which were novel gravitropic genes and the remaining were previously reported gravitropic genes. The identification of these genes provides deep insight into the molecular components of gravity sensing/signaling, and our screening system opens the possibility for new forward genetics in gravitropism.

## Materials and Methods

### Plant Materials and Growing Conditions

*Arabidopsis* seeds were sterilized, using 5% sodium hypochlorite and 1% Triton X-100, and then were sown on plates (AW2000; Eiken Chemical, Co., Ltd, Tokyo, Japan) with Murashige and Skoog (MS) medium that contained 1% [w/v] sucrose, 0.01% [w/v] myo-inositol, 0.05% MES-KOH (pH 5.7), and 0.05% [w/v] gellan gum. For the hypergravity condition, seeds were sown on plates (S01F04S; STEM, Co., Tokyo, Japan) with the MS medium that contained 0.03% [w/v] gelrite, instead of gellan gum. The seeds were vernalized at 4°C for 3 days, germinated at 23°C under continuous light, and then grown on the gel surface of vertical plates.

### T-DNA Insertion Lines, Transgenic Lines, and *ene* Single Mutant Lines

*abi4-1* and two T-DNA insertion lines, *eif6b* (SALK_139209) and *atcambp25* (SALK_005722C), were obtained from Arabidopsis Biological Resource Center (ABRC).

For a transformation-rescue experiment, we generated the *ARG1* construct (*pARG1::ARG1*), a KpnI-XhoI fragment of the genomic TAC K20N04 (∼8 kb), was cloned (Liu et al., 1999) and ligated into pENTR1A Gateway entry vector. The final construct was obtained by LR reaction in pFAST-R01 destination binary vector (Shimada et al., 2010). Transgenic seeds were generated by floral dip and were selected on MS medium supplied with hygromycin.

We crossed *eal1 ene* mutants with wild-type (Col) to generate *ene* single mutants. F2 seedlings were genotyped at two loci, *eal1* and *ene* mutation site, using SNP markers (See below; Filtering SNPs and predicting causative genes). We considered individuals which are homozygous wild-type at the *eal1* locus and homozygous mutant-type at the *ene* locus as *ene* single mutants.

### Mutagenesis and Screening Using Hypergravity

**FIGURE 1:**
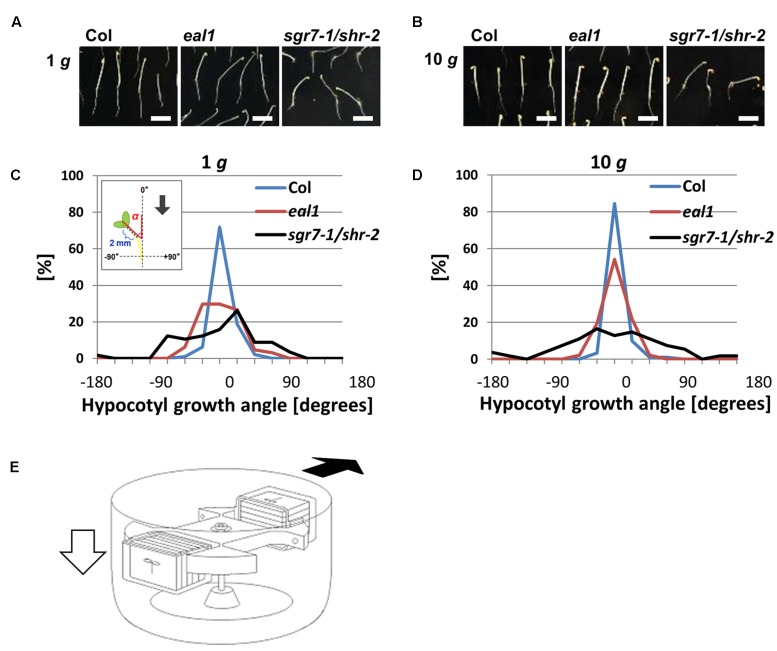
**Phenotypes of *eal1* and *sgr7-1/shr-2***(A)**, **(B)** 2-day-old Col, *eal1*, and *sgr7-1/shr-2*.** Etiolated hypocotyls were grown under 1 g **(A)** or 10 g **(B)** in the dark. All scale bars indicate 5 mm. **(C**,**D)** Distributions of angles in Col, *eal1*, and *sgr7-1/shr-2* (X-axis, angle; Y-axis, percentage), under 1 g (**C**: *n* ≥ 57) and 10 g conditions (**D**: *n* ≥ 54). The inset denotes a schematic diagram of hypocotyl angles. Growth angle (α) was measured between hypocotyl tip (∼2 mm) and the vertical axis. Arrow indicates the gravity vector. **(E)** A schematic diagram of mutant screening using a centrifuge. White arrow indicates the gravity vector (1 g before starting rotation/centrifugation). Black arrow indicates the hypergravity vector (10 g after starting rotation/centrifugation).

Approximately, 7000 seeds were mutagenized using 0.4% [w/v] EMS for 10 h. The M1 seeds were divided into 144 batches (∼50 seeds per batch). The M2 seeds were sown on plates for hypergravity conditions as mentioned above, which were set in swinging buckets (TS-4TB; Tomy Digital Biology, Co., Ltd, Tokyo, Japan) in a centrifuge (LX-141; Tomy Digital Biology, Co., Ltd, Tokyo, Japan; **Figure 1E**) and then centrifuged at 10 g [200 revolution per minute (rpm)] at 23°C in darkness for 2 days. Hypocotyls that were inclined over ±30° toward the direction of the hypergravity 10 g (0°), were selected (first screening), and their offspring (M3) seed was obtained. Approximately, 50 M3 seedlings from each mutant candidate were examined, using the experimental conditions described above, and their agravitropic phenotypes were confirmed (second screening). Subsequently, M3 seedlings were grown in the light for 45 h and in the dark for 24 h, and after being exposed to unilateral irradiation with blue light for 29 h, their phototropism was investigated (third screening).

### Analysis of Gravitropism in Hypocotyls and Roots

All the gravitropic angles and growth rates (i.e., root elongation length) were analyzed using ImageJ^1^. In 3-day-old etiolated hypocotyls, we measured the gravitropic angle formed between the growing direction of 2-mm hypocotyl tip and the vertical axis (**Figure 1C**, inset; α: hypocotyl growth angle), whereas in 3-day-old etiolated roots, we rotated the roots 90°, kept them in the dark for 12 h, and then measured primary root tip angles and root length (**Figure 3A**; 𝜃: bending curvature after reorientation of primary roots).

### Whole Genome Sequencing and SNP Detection

The *eal1 ene1–6* plants were backcrossed to the parental *eal1* plants, and the F2 population was screened under hypergravity conditions, as described above. Approximately, 80–300 individuals of the *eal1 ene* homozygous mutants and the *eal1* control mutant were pooled and used to prepare DNA libraries, as reported previously (Uchida et al., 2011). The DNA libraries for all seven lines were sequenced, using Illumina Genome Analyzer IIx (75 bp single read). The resulting sequences were aligned, and SNPs were detected using Strand NGS ver. 2.1 (Strand Scientific Intelligence, Inc.^2^; **Supplementary Table S1**).

### Filtering SNPs and Predicting Causative Genes

To identify line-specific SNPs, we compared the SNPs in each line to those detected in the other six lines and excluded a SNP when both the position and the base corresponded. Using the remaining SNPs, we searched for SNP-accumulating areas among the chromosomes (**Supplementary Figure S2**). These SNPs were also used as PCR markers (**Supplementary Table S2**) for mapping and confirmation of *ene* single mutants. Further, to predict the causative genes of the *ene*, synonymous SNPs and SNPs that were located in the intergenic regions or transposable elements were eliminated.

## Results

### New Screening System Using Hypergravity

When *Arabidopsis* seedlings are grown on the surface of a vertical agar plate, wild-type hypocotyls grow upward along the gravity vector, whereas the hypocotyls of gravitropic mutants grow in random directions. We measured angles (α) between the gravity vector (1 g) and growth direction in wild-type (Col), *eal1*, and *sgr7-1/shr-2* seedlings (**Figures 1A,C**). Variance in growth angle was larger in *sgr7-1/shr-2* and *eal1* mutants than in wild-type plants, confirming that *sgr7-1/shr-2* and *eal1* were gravitropic mutants at 1 g (**Table 1**). To apply hypergravity to *Arabidopsis* seedlings, we set up a light-tight centrifuge in which 50–100 seedlings were continuously exposed for 2 days (**Figures 1B,D,E**; for detail, see Materials and Methods). Although, the variance of *sgr7-1/shr-2* was not reduced at 10 g, likely owing to its lack of endodermis, that of *eal1* was reduced (**Table 1**), which indicated that *eal1* mutants retained gravity sensing machinery and that gravity sensitivity was restored. In the present study, we used the hypergravity condition (10 g) rather than the normal gravitational condition (1 g) to eliminate screening errors and to isolate *eal1* enhancers.

**Table 1:** Variancein the hypocotyl growth angles in Col, *eal1*, and *sgr7/shr-2.*

Line	*SD*		*F*-test (1 g vs. 10 g)
		
	1 g	10 g	
Col	12.998 (*n* = 96)	13.806 (*n* = 90)	0.5629
*eal1*	26.326 (*n* = 64)	17.556 (*n* = 92)	0.0004^∗^
*sgr7/shr-2*	50.141 (*n* = 57)	68.176 (*n* = 54)	0.0246^∗^


*^∗^A significant difference between 1 g and 10 g (*P* < 0.05).*

### Isolation of *eal1* Enhancer Mutants

To find second mutations in different genes that attenuate gravity sensitivity in *eal1*, *eal1* seeds were mutagenized with EMS. The resultant 57,600 M2 individuals were exposed to 10 g in the new screening system, and we selected 651 M2 candidates, hypocotyls of which were inclined over 30° to the gravity vector (**Table 2**; first screening). After obtaining the offspring of the M2 candidates, 50 individuals for each line were examined under the same screening condition and 181 M3 candidates were chosen (**Table 2**; second screening). Since this pool of M3 candidates should include mutants defective in not only gravitropism but also growth, we tested their phototropism as third screening. Finally, we isolated 12 candidates that exhibited normal phototropism (growth) but extremely reduced gravitropism at 10 g.

**Table 2:** Screening process for *eal1 ene* double mutants.

Flow	Number
M1 (EMS treatment)	7,000 seeds
↓ Self-fertilization	
M2	57,600 plants
↓ First screening	651 lines
↓ Self-fertilization	
M3	
↓ Second screening	181 lines
↓ Third screening	12 lines
↓ Self-fertilization	
*eal1 ene*	12 lines


We named the *eal1* enhancer lines *eal1 ene* (*enhancer of eal1*) and analyzed six of the 12 lines (*eal1 ene1–6*). Accordingly, *eal1 ene1–6* were backcrossed with the parental *eal1* line, and the gravitropism of the F2 population was investigated at 10 g. The segregation ratio of wild-type to mutant phenotypes was 3:1 (**Table 3**), which indicated that each *eal1 ene* was the result of a single recessive mutation.

**Table 3:** Genetic analysis of *ene.*

Cross	Number of F2 plants			Expected ratio	χ^2^
			
	Total	WT-like	Inclined (over ± 30°)		
*eal1* × *eal1 ene1*	89	71	18	3:1	1.08^∗^
*eal1* × *eal1 ene2*	84	68	16	3:1	1.59^∗^
*eal1* × *eal1 ene3*	80	65	15	3:1	1.67^∗^
*eal1* × *eal1 ene4*	100	79	21	3:1	0.85^∗^
*eal1* × *eal1 ene5*	98	77	21	3:1	0.67^∗^
*eal1* × *eal1 ene6*	97	78	19	3:1	1.52^∗^


*^∗^No significant difference between the ratio and the expected (*P* > 0.05).*

### Phenotypes of *eal1 ene* Mutants

**FIGURE 2:**
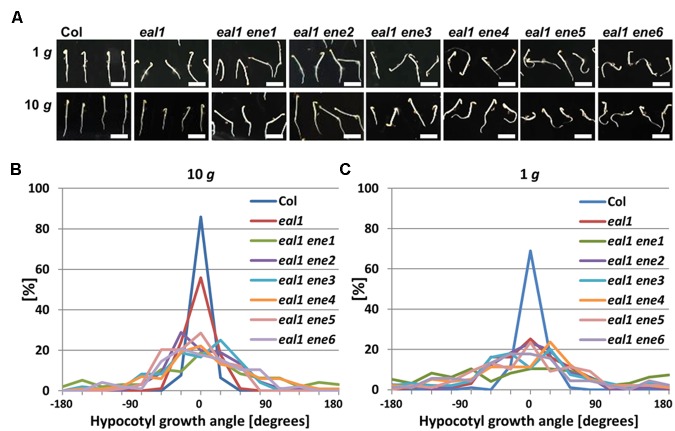
**Quantification of hypocotyl growth angles in *eal1 ene* double mutants.** **(A)** 3-day-old Col, *eal1*, and *eal1 ene* etiolated hypocotyls grown under 1 or 10 g. All scale bars indicate 5 mm. **(B**,**C)** Distributions of angles in Col, *eal1*, and *eal1 ene* hypocotyls grown under 10 (**B**: *n* ≥ 90) and 1 g conditions (**C**: *n* ≥ 82).

We quantified the phenotype of *eal1 ene* hypocotyls under 1 g and hypergravity conditions (**Figures 2A,C**). The variance of growth angle was significantly higher in hypocotyls of the *eal1 ene1–6* mutants than in those of *eal1* at 10 g (**Figure 2B**), whereas no significant difference was observed at 1 g (**Figure 2C**).

**FIGURE 3:**
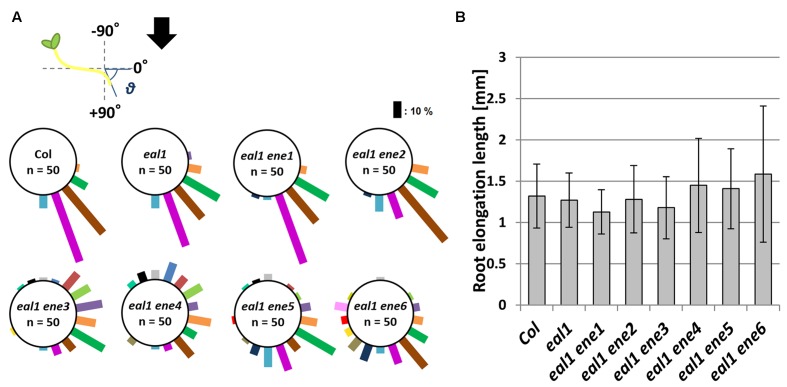
**Root gravitropism in *eal1 ene* double mutants.** **(A)** Measuring the angle (𝜃) from primary root tip to the horizontal axis. Arrow indicates the gravity vector. 3-day-old etiolated seedlings were used. The bar length is proportional to the number of plants observed in each 20° bin. **(B)** The root extension length in each line. Error bar shows standard deviation (SD). No significant difference was observed between *eal1* and each *eal1 ene* (Student’s *t*-test, *P* < 0.01).

Since some *eal1 ene* mutants showed agravitropic responses in their roots (**Figure 2A**), we quantified growth rate and gravitropism in all the *eal1 ene* mutants. We found that root gravitropism was more random in *eal1 ene3*–*6* roots than that in *eal1* roots (**Figure 3A**). Meanwhile, *eal1 ene2* plants exhibited slightly reduced gravitropism, and the phenotype of *eal1 ene1* was identical to that of *eal1* and wild-type plants. There was no statistical difference in the growth rate among wild-type, *eal1*, and *eal1 ene1–6* plants (**Figure 3B**), and the reduced or agravitropic responses resulted from the *ene2–6* mutations, since the parental *eal1* mutants exhibited normal gravitropism in primary roots (Fujihira et al., 2000; Morita et al., 2007). Thus, *ENE2*–*6* play a role in genetic pathways for both shoot and root gravitropism, whereas *ENE1* is only involved in shoot gravitropism.

We also observed morphology, such as leaf shape and lateral organs, in the *eal1* enhancer lines at different ages. The morphologies of most of the aerial parts of 12-day-old *eal1 ene* seedlings were identical to those of the aerial parts of wild-type and *eal1* seedlings (**Supplementary Figure S1A**), and the inflorescence stems of 4-week-old *eal1 ene1–6* mutants exhibited gravitropism that was similar to that of *eal1* seedlings at 1 g (**Supplementary Figure S1B**). Since the inflorescence stems in *eal1* do not exhibit gravitropism at 1 g (Fujihira et al., 2000; Morita et al., 2007), we assumed that the phenotype was caused by the *eal1* mutation. These data support the conclusion that *ene1–6* have no obvious phenotypes, other than the gravitropism defect.

### *ENE3–6* were Revealed by Whole Genome Sequencing and SNP Analysis

Since EMS treatment causes thousands of SNPs in *Arabidopsis* chromosomes, we needed to eliminate irrelevant SNPs in order to identify *ene* mutations, using next-generation sequencing (NGS). Thus, we backcrossed *eal1 ene1–6* with the parental *eal1* line and used 80–300 F2 individuals that exhibited the agravitropic response at 10 g for NGS. Because the EMS-derived SNPs in *ene* lines are supposed to accumulate near *ene* mutations, rather than at other chromosomal sites, the frequency of SNPs was examined throughout the chromosomes of each *eal1 ene* mutant (**Supplementary Figure S2**).

Our NGS data revealed several regions with high SNP frequencies (**Table 4**; **Supplementary Figure S2**), so we targeted these regions to investigate SNPs and candidate genes. One SNP, in *ene3*, was located at a splice acceptor site in intron 10 of the *ALTERED RESPONSE TO GRAVITY 1* (*ARG1*) gene (2255-1C > T), which is important for hypocotyl and root gravitropism (Fukaki et al., 1997; Harrison and Masson, 2008; Kumar et al., 2008). Therefore, the SNP in *ene3* could affect the splicing process of *ARG1* and gravitropism. In *ene4*, we found a SNP in exon 8 of the *AUXIN RESISTANT 1* (*AUX1*) gene (Gly381Asp), which encodes an auxin influx carrier that is necessary for root gravitropism (Marchant et al., 1999). The SNP was located in a transmembrane domain, which is a new allele of *AUX1* (Swarup et al., 2004). In *ene5*, we found a SNP in a lariat intron of the *PIN-FORMED 2* (*PIN2*) gene (3128-17A > G), which encodes an auxin efflux carrier that is necessary for root gravitropism (Chen et al., 1998; Luschnig et al., 1998; Müller et al., 1998; Utsuno et al., 1998), suggesting that the mutation affects both *PIN2* splicing and auxin distribution during gravitropism. In *ene6*, we found a SNP in exon 2 of the *PIN2* gene (Pro111Leu). This amino acid substitution was in a transmembrane domain that could be critical for auxin transport. Taken together, our findings of these three genes (*ARG1, AUX1*, and *PIN2*) demonstrate the utility of our hypergravity screening system and SNP-based method for isolating and characterizing enhancer mutants in gravitropism, which we used to identify four novel alleles.

**Table 4:** Prediction of causative genes for *ene* using next-generation sequencing and SNP-based markers.

		*ene1*	*ene2*	*ene3*	*ene4*	*ene5*	*ene6*
Number of reads		41,074,711	64,100,951	68,227,748	19,764,902	20,610,570	40,856,830

Number of SNP	Raw	59,953	64,703	62,328	34,372	36,018	43,110
	
	Extracted	194	141	89	105	178	65

SNP-accumulation area (Region)		Latter half of Chr 2 (over 13,000,000)	First half of Chr 5 (under 13,500,000)	Latter half of Chr 1 (over 15,000,000)	Latter half of Chr2 (over 10,000,000)	Lower end of Chr5 (over 18,000,000)	Lower end of Chr5 (over 18,000,000)

Number of candidate genes		35	25	21	14	36	27

Gravitropism-related gene (AGI code, gene, Homozygous SNP frequency)		NA	NA	AT1G68370, *ARG1*, 66.04%	AT2G38120, *AUX1*, 84.62%	AT5G57090, *PIN2*, 87.5%	AT5G57090, *PIN2*, 81.48%


### *ENE3* is an Allele of *ARG1*

**FIGURE 4:**
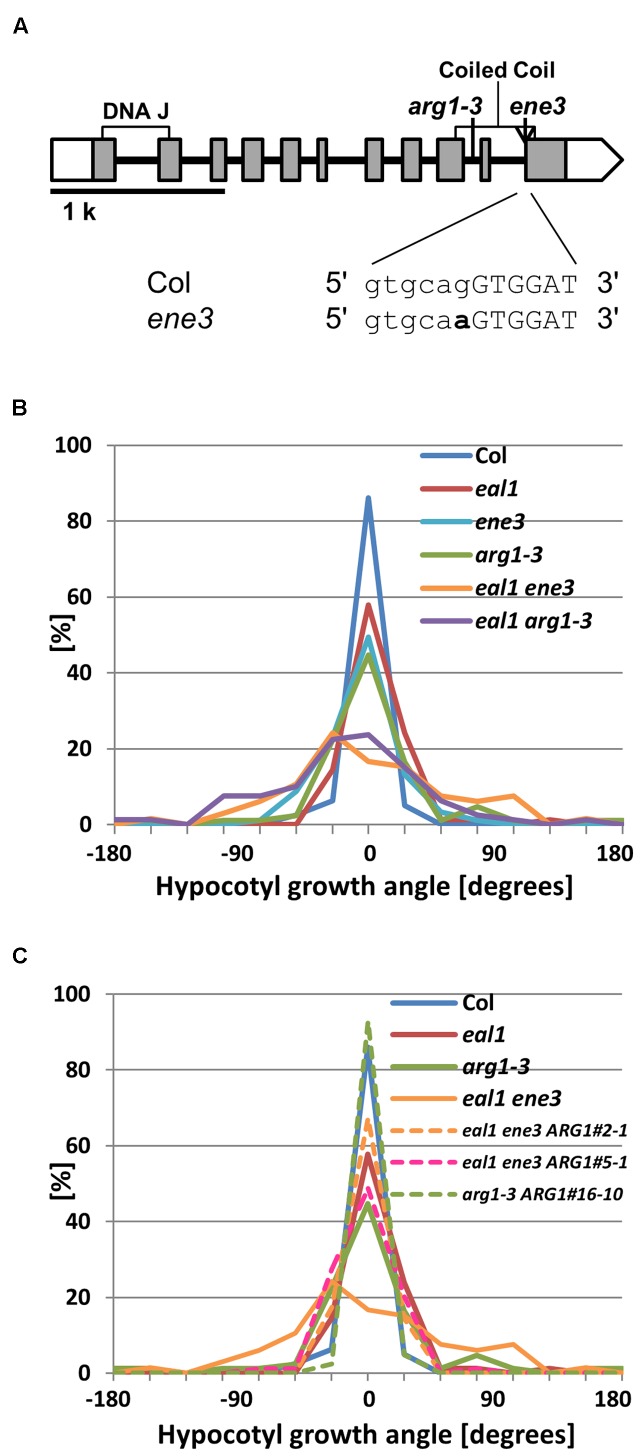
**(A)** *ARG1* gene structure. White and shaded boxes indicate UTRs and coding regions, respectively. Black bold lines indicate introns. Two regions of the gene encoding DNA J and coiled coil domains within the protein are shown by open rectangles underneath the gene exons. At the bottom, bold letter indicates *ene3* mutation. **(B)** Quantification of hypocotyl growth angles in *ene3* single and the double mutants under 10 g (*n* ≥ 66). **(C)** Transformation-rescue experiment (*n* ≥ 66).

A SNP was located at a splice site in *ARG1* in *ene3* (**Figure 4A**). We crossed *arg1–3*, a line with a mutant allele of *ARG1*, with *eal1* mutant to investigate whether *arg1* mutation enhances *eal1* phenotype in gravitropism (Harrison and Masson, 2008). The *eal1 arg1–3* double mutant showed similar variance to that of *eal1 ene3*, whereas *arg1–3* single mutant showed similar variance to that of *eal1* (**Figure 4B**). This data indicates that *arg1* mutation enhances *eal1* gravitropism. Further, we obtained the *ene3* single mutant and compared its gravitropism to the *arg1–3* single mutant under 10 g. Since both single mutants showed *eal1*-like variances (**Figure 4B**), each allele has similar enhanced effects on *eal1* gravitropism. We also generated transgenic lines carrying a *pARG1::ARG1* in *arg1–3* or *eal1 ene3* background and conducted a transformation-rescue experiment. Although, single *arg1–3* mutant showed large variance under 10 g condition, a transgenic line, *arg1–3 ARG1#16-10*, showed wild-type variance (**Figure 4C**) indicating that this construct functions properly. Two independent lines, *eal1 ene3 ARG1#2-1* and *#5-1*, showed *eal1*-like variances in hypocotyl gravitropism (**Figure 4C**). Since *ARG1* rescues the gravitropism defect in *eal1 ene3*, *ene3* is a mutation in *ARG1*.

### *ENE4* is an Allele of *AUX1*

**FIGURE 5:**
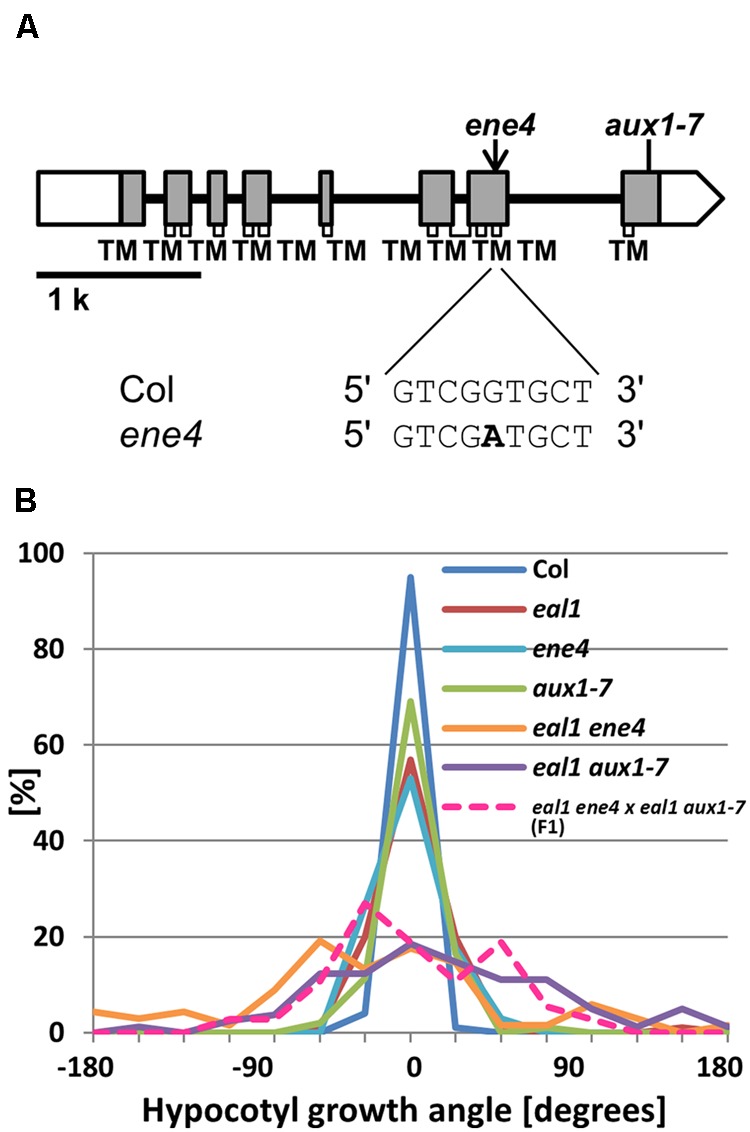
**(A)** *AUX1* gene structure. White and shaded boxes indicate UTRs and coding regions, respectively. Black bold lines indicate introns. Regions of the gene encoding transmembrane domains (TMs) within the protein are shown by open rectangles underneath the gene exons. At the bottom, bold letter indicates *ene4* mutation. **(B)** Quantification of hypocotyl growth angles in *ene4* single and the double mutants under 10 g, along with F1 progeny from a cross between *eal1 ene4* and *eal1 aux1–7* plants (*n* ≥ 37).

In *ene4*, a SNP was found in a region of *AUX1* that encodes a transmembrane domain within the corresponding proteins (**Figure 5A**). We generated *eal1 aux1–7* double mutants to confirm whether a mutation in *AUX1* can enhance defective gravitropism in *eal1* (Pickett et al., 1990). Under 10 g, the variance of growth angle in *eal1 aux1–7* was similar to that in *eal1 ene4* (**Figure 5B**). The variance in *aux1–7* was similar to that in *eal1*, indicating that *aux1*–7 mutation enhances *eal1* gravitropism. Further, we obtained *ene4* single mutant and compared it to *aux1*–7 single mutant. Single mutants of *ene4* or *aux1–7* show *eal1*-like variances, therefore these mutations have similar enhanced effects on *eal1* gravitropism. We conducted an allelism test to determine whether *ene4* and *aux1–7* are allelic by crossing *eal1 ene4* and *eal1 aux1–7* (**Figure 5B**). The variance of F1 hypocotyls was identical to that in *eal1 ene4* and *eal1 aux1–7*. These data demonstrate that *ene4* is a mutation in *AUX1* and is a new allele of *AUX1*.

### *ENE5* and *ENE6* are Alleles of *PIN2*

**FIGURE 6:**
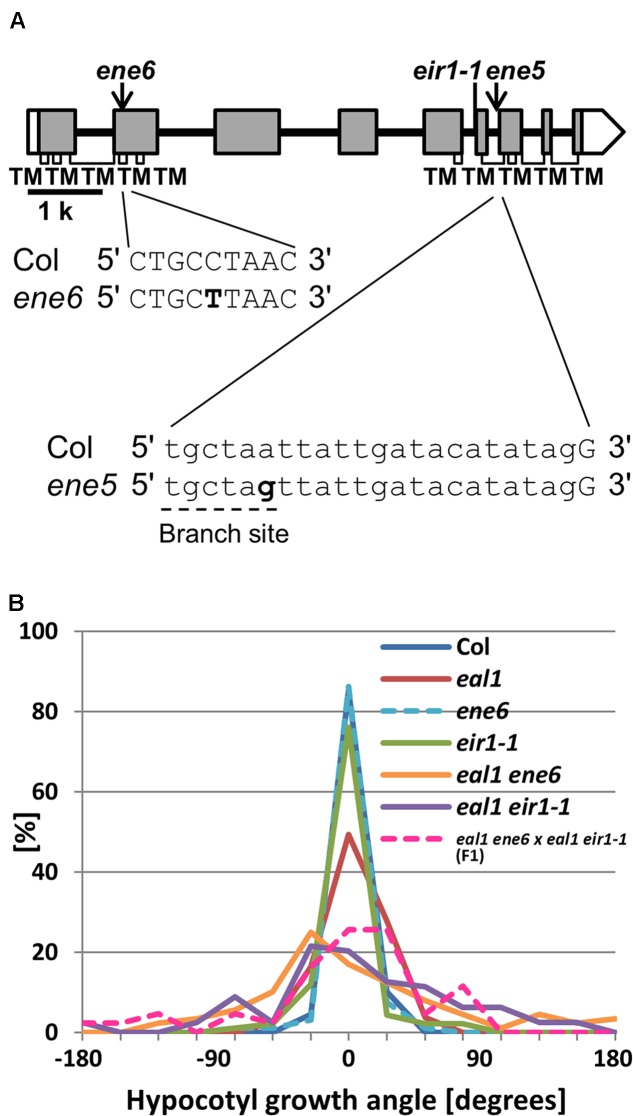
**(A)** *PIN2* gene structure. White and shaded boxes indicate UTRs and coding regions, respectively. Black bold lines indicate introns. Regions of the gene encoding TMs with in the protein are shown by open rectangles underneath the gene exons. At the bottom, bold letters indicate *ene5* and *ene6* mutations. A dashed line indicates the branch site for lariat structure. **(B)** Quantification of hypocotyl growth angles in *ene6* single and the double mutants under 10 g, along with F1 progeny from a cross between *eal1 ene6* and *eal1 eir1-1* plants (*n* ≥ 43).

Both lines *ene5* and *ene6* had a SNP located in *PIN2* (**Figure 6A**). We generated *eal1 eir1-1* double mutant and compared it to *eal1 ene5* and *eal1 ene6* under 10 g (**Supplementary Figure S3**). Since these variances were similar, *ene5* and *ene6* were suggested to be allele of *PIN2*. We obtained *ene5* and *ene6* single mutants and then compared to *eir1-1* single mutant. All three showed wild-type variances. This indicates that these three mutations can enhance *eal1* gravitropic defect. To determine whether *ene5* and *ene6* are in a same gene, we crossed *eal1 ene5* to *eal1 ene6* and then conducted an allelism test under 10 g (**Supplementary Figure S3**). The F1 mutants showed a variance similar to those of *eal1 ene5* and *eal1 ene6* therefore *ene5* and *ene6* are alleles of a same gene. We focused further on *ene6* by crossing *eal1 ene6* to *eal1 eir1-1* for a second allelism test (**Figure 6B**). In F1 mutants, the variance was identical to that in parental *eal1 ene6* and *eal1 eir1-1* mutants. We revealed that both *ene5* and *ene6* are mutations in *PIN2*.

### Mapping of *ene1* Using SNP Markers

No known gravitropism-related genes were found in the targeted regions of the *eal1 ene1* and *eal1 ene2* mutants (**Table 4**). Therefore, the causative genes of *ene1* and *ene2* phenotypes should be novel. In *eal1 ene2*, we found SNP-accumulation areas at the center of chromosome 1 and in the first half of chromosome 5 (**Supplementary Figure S2**). Since all the SNPs in chromosome 1 were located in intergenic regions, we concluded that the causative gene of *ene2* was located in the first half of chromosome 5 (**Table 4**). Meanwhile, we found 25 EMS-derived SNPs within 13.5 Mbp, in the first half of chromosome 5, which contains 25 genes (**Table 4**).

**FIGURE 7:**
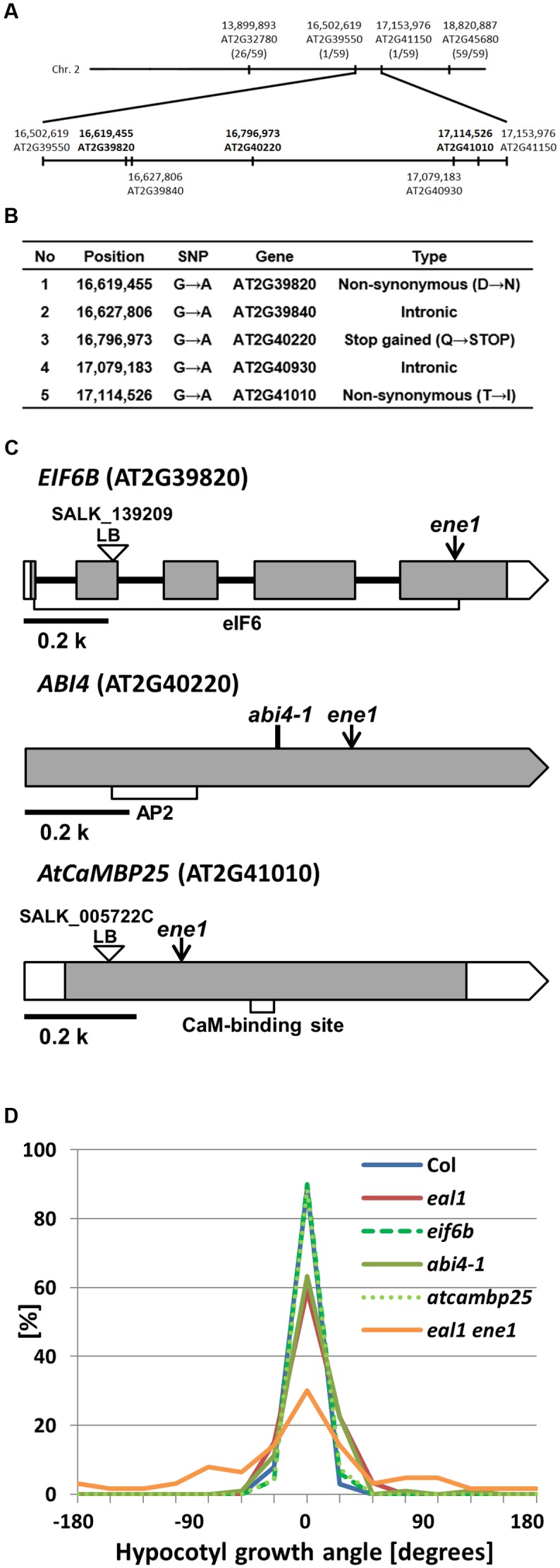
**Mapping of *eal1 ene1* using SNP-based markers.** **(A)** Genomic locations of the PCR markers developed from the detected SNPs. Upper row shows chromosome 2. The positions of four PCR markers are indicated, with the number of recombinants. The *ene1* mutation was *(mapped on the latter half of chromosome 2, between markers AT2G39550 (16,502,619 bp) and AT2G41150 (17,153,976 bp). The candidate genes (five SNPs) for *ene1* mutants are shown on the bottom. Bold letters indicate SNPs in the exon regions. **(B)** Annotation of five SNPs in the defined region. **(C)***EIF6B*, *ABI4*, and *AtCaMBP25* gene structures. White and shaded boxes indicate UTRs and coding regions, respectively. Black bold lines indicate introns. eIF6, APETALA 2 (AP2), and calmodulin binding domains (CaM-binding site) are shown by open rectangles under the corresponding gene exons in *EIF6B*, *ABI4*, and *AtCaMBP25*, respectively. Arrows indicate the position of SNPs identified in *ene1*. **(D)** Quantification of hypocotyl growth angles in single mutants under 10 g (*n* ≥ 63).)*

On the other hand, for *ene1*, we identified 35 SNPs within 6.2 Mbp, in the latter half of chromosome 2. To identify the causative gene of *ene1*, we designed new PCR markers that recognize the EMS-derived SNPs and performed map-based cloning (**Figure 7A**; **Supplementary Table S2**). Consequently, the responsible SNP was predicted to occur in a ∼650 kbp region between 16,502,619 (AT2G39550) and 17,153,976 (AT2G41150), in which there were five SNPs (**Figure 7B**). Two SNPs, 16,627,806 (AT2G39840) and 17,079,183 (AT2G40930), were located in introns and did not associate with any splice site or lariat formation. Two other SNPs, 16,619,455 (AT2G39820) and 17,114,526 (AT2G41010), caused amino acid substitutions. The former SNP (Asp203Asn) was located in exon 5 of *EIF6B* (AT2G39820), which encodes the eukaryotic initiation factor 6 domain (*eIF6*), whereas the latter SNP (Thr69Ile) was located in exon 1 of *AtCaMBP25* (AT2G41010), which encodes a calmodulin binding protein. The last SNP creates a stop codon (Gln205X) at 16,796,973 (AT2G40220) in exon 1 of *ABI4* (AT2G40220), which encodes a member of DREB subfamily A-3 of ERF/AP2 transcription factor family. We examined gravitropism in three single mutants of *EIF6B*, *ABI4*, and *AtCaMBP25* (**Figures 7C,D**). Both SALK lines, *eif6b* and *atcambp25*, showed small variances, which are comparable to wild-type. However, the *abi4-1* single mutant had a large variance as compared to wild-type suggesting that *ENE1* is likely to be *ABI4.* This gene has not been reported in association with gravitropism so far. Identification of *ENE1* and *ENE2* will contribute to further understanding of the mechanism of gravity sensing in *Arabidopsis* hypocotyls.

## Discussion

A variety of gravitropic mutants have been reported in *Arabidopsis*, but their enhancer/suppressor mutants have not been isolated under different gravitational conditions. Using our new screening system and NGS in combination with SNP-based markers, we isolated six *eal1 ene* mutants with enhanced gravitropism defect. Single and double mutant analyses revealed that three genes responsible are known to be related to gravitropism (*ARG1*, *AUX1*, and *PIN2*). The causative genes for *ene3–6* phenotypes were successfully predicted by SNP analyses. We expect that remaining two genes are novel.

### *ENE3* is an Allele of *ARG1*

The gene *ARG1* is recognized to be important for root and hypocotyl gravitropism (Sedbrook et al., 1999; Boonsirichai et al., 2003) and localization of the auxin efflux carrier PIN3 in columella cells, which are gravity-sensing cells in roots (Harrison and Masson, 2008). The SNP in *ARG1* was found in a canonical splice site (GT-AG) in intron 10. Thus, the mutation likely causes abnormal splicing patterns, resulting in pre-mature stop codon in intron by no splicing or in the fifth amino acid in exon 11 by splicing with shifted one base toward the 5′. The ARG1 protein contains a DnaJ-like domain at its N terminus, a putative transmembrane region, and a coiled-coil domain at its C terminus (Sedbrook et al., 1999; Guan et al., 2003). The *ene3* mutation located in the coiled-coil region may affect protein-protein interactions.

Single *arg1* mutants exhibit decreased gravitropism in their hypocotyls and roots but normal phototropism in their hypocotyls (Sedbrook et al., 1999; Guan et al., 2003), which is similar to the *eal1 ene3* phenotype (**Figures 2** and **3A**; **Table 1**; third screening), thus, supporting the idea that *ene3* is a mutation in *ARG1* and represents a new allele. Further, single and double mutant analysis and a transformation-rescue experiment revealed that the *eal1* phenotypes are enhanced by the *ene3* mutation in *ARG1* (**Figure 4B**), which indicates that *ARG1* and *EAL1* regulate gravitropism independently. It has been reported that ARG1 proteins in endodermal cells function in hypocotyl gravitropism (Boonsirichai et al., 2003). In the *eal1* mutant, a single amino acid deletion (E230X) occurs in the transcription factor SGR7/SHR (Morita et al., 2007). Therefore, in the endodermis, it is likely that the expressions of many gravitropism-related genes, such as *SCARECROW* (*SCR*), are downregulated. Nevertheless, because the *ene3/arg1* mutation enhances the *eal1* phenotypes, the expression of *ARG1* may be regulated in a SHR-SCR independent manner.

### *AUX1* Plays a Role in Hypocotyl Gravitropism

In the *eal1 ene4* mutant, we found a mutation in *AUX1*, which encodes an auxin influx carrier protein that plays a role in differential growth during gravitropism through the modulation of polar auxin transport (Michniewicz et al., 2007). In *AUX1*, 11 transmembrane regions were predicted by the PRED-TMR2 software, and the SNP (Gly381Asp) was identified in the 10th transmembrane region.

Single *aux1* mutants exhibit reduced gravitropism in primary roots (Marchant et al., 1999; Swarup et al., 2004), and normal phototropism in hypocotyls (Okada and Shimura, 1992; Watahiki et al., 1999). In *eal1 ene4* mutants, primary root gravitropism and hypocotyl phototropism are similar to single *aux1* mutants, suggesting that *ENE4* is identical to *AUX1*. A previous study mentioned that AUX1 functions as a modulator of gravitropism defect in *arf7* hypocotyls (data not shown in Stone et al., 2008). Here we explicitly confirm the contribution of AUX1 to hypocotyl gravitropism.

### PIN2 Functions in Hypocotyl Gravitropism

PIN2 is an auxin efflux carrier that is critical for differential growth during gravitropism, through its modulation of polar auxin transport (Chen et al., 1998; Luschnig et al., 1998; Müller et al., 1998; Utsuno et al., 1998). In both *eal1 ene5* and *eal1 ene6*, we identified different point mutations in *PIN2* (**Table 4**). PIN2 has 10 transmembrane regions (Müller et al., 1998) and the SNP (3128-17A > G) is located at the branch site for lariat structure within a region of the gene that encodes the seventh transmembrane domain, which suggests that a stop codon is generated in the intron. For the *ene6* mutant, amino acid substitution (Pro111Leu) occurs in the third transmembrane region and should affect the function of auxin transport.

According to previous reports, single *pin2* mutants exhibit decreased gravitropism in roots (Chen et al., 1998; Luschnig et al., 1998; Müller et al., 1998; Utsuno et al., 1998) and normal phototropism in hypocotyls (Wan et al., 2012). Both *eal1 ene5* and *eal1 ene6* mutants exhibit *pin2* mutant-like phenotypes regarding the gravitropism in primary roots and the phototropism in hypocotyls. Our double mutant analysis and allelism test revealed that *ene5* and *ene6* are different alleles of *PIN2* (**Supplementary Figure S3**; **Figure 6B**). *pin2* hypocotyls are supposed to show a transient defect in gravitropism, although the data is not shown (E. R. and Kenneth L. Poff, unpublished results in Chen et al., 1998). Further, in 5-day-old etiolated hypocotyls, *PIN2* is expressed in roots and also in shoots including hypocotyls and cotyledons (Chen et al., 1998). These results indicate that PIN2 functions in hypocotyl gravitropism, owing to overlap with the *eal1* background.

### *ENE1* is a Novel Gene Related to Gravitropism in Hypocotyls

In the present study, we identified five possible mutations in the *ene1* mutant, in a genomic region without any previously reported gravitropism-related genes (**Figure 7B**). Thus, the gene affected by the *ene1* mutation is a novel gravitropism-related gene. Among the genes found in the region, *ABI4* is known to play a role in sensitivity to abscisic acid during seed germination (Finkelstein, 1994) and is highly expressed in seeds and weakly expressed in hypocotyls and shoots (Finkelstein et al., 1998; Söderman et al., 2000). Previous studies have also suggested that ABI4 functions as a transcription factor (Finkelstein et al., 1998; Gregorio et al., 2014). The *eal1 ene1* mutant has a mutation in *ABI4* that generates a stop codon (Gln205X) in a transcriptional activation region. The expression levels of the downstream factors of ABI4 may be altered, leading to reduced gravitropism in hypocotyls.

The other candidate mutations were found in *AtCaMBP25* and *EIF6B*. AtCaMBP25 is a calmodulin binding protein that is induced in response to various environmental stresses, such as drought, low temperature, and salt stress (Perruc et al., 2004). Due to the mutation in *AtCaMBP25*, the amino acid at position 69 was changed from hydrophilic threonine to hydrophobic isoleucine. Since it is known that cytosolic calcium signal and calmodulin network is important for gravity sensing in hypocotyls (Toyota and Gilroy, 2013; Toyota et al., 2013a) and roots (Sinclair and Trewavas, 1997), AtCaMBP25 might be involved in a downstream signal in an early gravitropic response. Meanwhile, eIF6 is a eukaryotic initiation factor concerned with the formation of the ribosome complex, and has two homologs in *Arabidopsis thaliana*: *EIF6A* and *EIF6B* (Guo et al., 2011; Moore et al., 2016). The eIF6 domain is composed of 206 amino acids. In this mutation, residue 201 is changed from aspartic acid to asparagine due to the mutation. This single amino acid substitution could affect the function of EIF6B as a translation initiator.

Although, the other possible mutations are located in introns of *TOPP4* (AT2G39840) and *AtUBP5* (AT2G40930) in the targeted region of *ene1*, these genes are unlikely to be responsible because the mutations do not occur in the splice site or the lariat site. In addition, only *abi4-1* mutants showed gravitropism defect under 10 g by the single mutant analysis (**Figure 7D**). We concluded that the causative gene of *ene1* might be *ABI4*, the function of which has not been clarified in gravitropism.

### Enhancer Mutations Can be Mapped Efficiently Using Hypergravity and Parental Crossing

The hypocotyls of *Arabidopsis eal1* mutants exhibit exceptionally weak gravitropism at 1 g. Thus, it is extremely difficult to isolate an enhancer mutant showing further reduced gravitropism at 1 g. In the present study, we used hypergravity to enhance the gravitropic response in *eal1*, which increased the efficiency of isolating enhancer lines. After identifying enhancer candidates, responsible genes were investigated using NGS. However, using SNP analysis to predict candidate genes has limitations, despite the adequate number of genomes and coverage (James et al., 2013). In conventional mapping, the F2 population is crossed with a different accession in which there is an abundance of polymorphic markers. Therefore, if used conventional methods to map *eal1* enhancer mutations, 1/16 of the F2 population would be homozygous for both *eal1* and *ene* mutations. In the present study, by crossing to the parental *eal1*, we expected 1/4 of the F2 population to be double mutant, *eal1 ene*. We developed new PCR markers based on SNPs that were identified by NGS and drastically reduced the number of candidate genes. Indeed, the mapping of *eal1 ene1* in 59 F2 mutants allowed us to narrow the region containing the causative *ene1* gene from ∼6.2 Mbp to 650 kbp and reduced the number of candidate genes from 35 to 5. We can apply this strategy to identify the causative gene of *ene3* as well. Our unique screening and mapping system enabled us to discover new enhancer lines and to provide insight into the gravity sensory machinery of plants.

## Author Contributions

MTM and MTo conceived and designed the study. TK advised on the experimental design of NGS. AM, MTo, MS, and MM performed experiments, and AM and MS analyzed NGS data. MTM, MTo, AM, and MTa interpreted data. AM wrote the manuscript and all authors reviewed and edited the manuscript.

## Conflict of Interest Statement

The authors declare that the research was conducted in the absence of any commercial or financial relationships that could be construed as a potential conflict of interest.

The reviewer PM declared a shared affiliation, though no other collaboration, with one of the authors MT to the handling Editor, who ensured that the process nevertheless met the standards of a fair and objective review.

## Footnotes

1 https://imagej.nih.gov/ij/

2 http://www.strand-ngs.com/
