# Supplementary material for: Isolation of New Gravitropic Mutants under Hypergravity Conditions
Source: Front Plant Sci. 2016 Sep 29;7:1443. doi: 10.3389/fpls.2016.01443 (PMC5040707; doi:10.3389/fpls.2016.01443)
Supplement: Supplementary file 1 [file Table_1.DOCX]

Supplementary Material

Isolation of new gravitropic mutants under hypergravity conditions

Akiko Mori, Masatsugu Toyota, Masayoshi Shimada, Mika Mekata, Tetsuya Kurata, Masao Tasaka and Miyo Terao Morita*

*** Correspondence:** Miyo Terao Morita: mimorita@agr.nagoya-u.ac.jp

# Supplementary Figures and Table

**Supplementary Figure 1** | **(A)** 10-day-old seedlings of Col, *eal1*, and *eal1 ene1‒6*. All scale bars indicate 1 cm. **(B)** 4-week-old inflorescence stems of Col, *eal1*, and *eal1 ene1‒6*. All scale bars show 5 cm.

**Supplementary Figure 2 |** SNP-accumulation areas in *eal1 ene1‒6* are shown (X-axis, chromosome regions; Y-axis, the SNP count per 500 kbp sliding window).

**Supplementary Figure 3 |** Quantification of hypocotyl growth angles in *ene5* and *ene6* single and the double mutants under 10 g (n ≥ 24).

**Supplementary Table 1 |** Procedure and modified parameters in Strand NGS

| 1. Tools – Options | | | | | | | |
| --- | --- | --- | --- | --- | --- | --- | --- |
|  | Configuration Dialog – DNA Variant Analysis – SNP Detection | | | | | | |
|  |  | | Ignore reads with base quality less than | | | 0 | |
| 1. [Workflow]→[Alignment]→[Run Alignment] | | | | | | | |
|  | | New Experiment window | | | | | |
|  |  |  | | Experiment Type | DNA Alignment ChiP-Seq/DNA-Seq | | |
|  |  |  |  | Workflow Type | Advanced Analysis | | |
|  |  | DNA Alignment ChiP-Seq/DNA-Seq, Choose Meta Data window | | | | | |
|  |  |  | | Organism | Arabidopsis | | |
|  |  |  |  | Build | Tair10 | | |
|  |  |  |  | Sequence Platform | Illumina | | |
|  |  |  |  | Library layout | Single End | | |
|  |  | Select Trimming Parameters window | | | | | |
|  |  |  | | Number of bases to trim from 3’ end | | | 0 |
|  |  |  |  | Number of bases to trim from 5’ end | | | 0 |
|  |  |  |  | Trim 3’ end with average quality less than | | | 10 |
| 1. [Workflow]→[Utilities]→[Create DNA Variant Analysis Experiment] | | | | | | | |
|  | | New Variant Analysis Experiment, Choose Data window | | | | | |
|  |  |  | | Gene Annotation | Ensemble Genes (2012.12.16) | | |
|  | | [Workflow]→[Analysis]→[Filtering] | | | | | |
|  | | Read quality metrics | | | | | |
|  | | Duplicate reads | | | | | |
| 1. [Workflow]→[Analysis]→[SNP detection] | | | | | | | |
|  | | Select Inputs window | | | | | |
|  |  |  | | Confidence score (-10*log10(p-value)) cutoff | | | 30 |
|  |  |  |  | Ignore reference locations with coverage below | | | 5 |
|  |  |  |  | Ignore reference locations with variants below | | | 2 |

**Supplementary Table 2 |** SNP markers for *ene* mutants

| *ene* | AGI code | Primer sequence | Restriction Enzyme |
| --- | --- | --- | --- |
| *ene1* | AT2G32780 | Forward: 5’-TAAGGCTGACTGCAGTGATG-3’  Reverse: 5’-GATAGCGGTCGAAAGAAAGG-3’ | DdeI |
|  | AT2G39550 | Forward: 5’-TGTCAGCATATAATGATTAAATAAGCCATG-3’  Reverse: 5’-GGTAGCTATTCCTTACCTGC-3’ | StyI |
|  | AT2G41150 | Forward: 5’-TTTCACTTCGGTGATCTCCACCGGCGGCTT-3’  Reverse: 5’-CAGTTCTTACCAGGACAATCG-3’ | DdeI |
|  | AT2G45680 | Forward: 5’-TTCAACAGGCTTCCACGATGG-3’  Reverse: 5’-AGCTTGAGCTCGGAGCCATAACCGAAGTCG-3’ | XmnI |
| *ene3* | AT1G68370  (*ARG1*) | Forward: 5’-GTTTGGTTTGTGTATATTGAATAATGCTGC-3’  Reverse: 5’-GATTCATCTCCCTGAGCTTTGC-3’ | PstI |
| *ene4* | AT2G38120  (*AUX1*) | Forward: 5’-TGGGATGCATGACACAAAGAGC-3’  Reverse: 5’-GATATAGACGGTGAAGCTAACAAGAAGAGCG-3’ | SalI |
| *ene5* | AT5G57090  (*PIN2*) | Forward: 5’-TGCTGGTCTTGGAATGGCTATG-3’  Reverse: 5’-TGCCATAAATAGACCTATATGTATCACTAG-3’ | SpeI |
| *ene6* |  | Forward: 5’-TGATAACGCTCTTTTCACTATCAACAAAGC-3’  Reverse: 5’-TCTGAAGCACCACGATCTGC-3’ | HindIII |
