# Supplementary figures and images for: Isolation of New Gravitropic Mutants under Hypergravity Conditions
Source: Front Plant Sci. 2016 Sep 29;7:1443. doi: 10.3389/fpls.2016.01443 (PMC5040707; doi:10.3389/fpls.2016.01443)

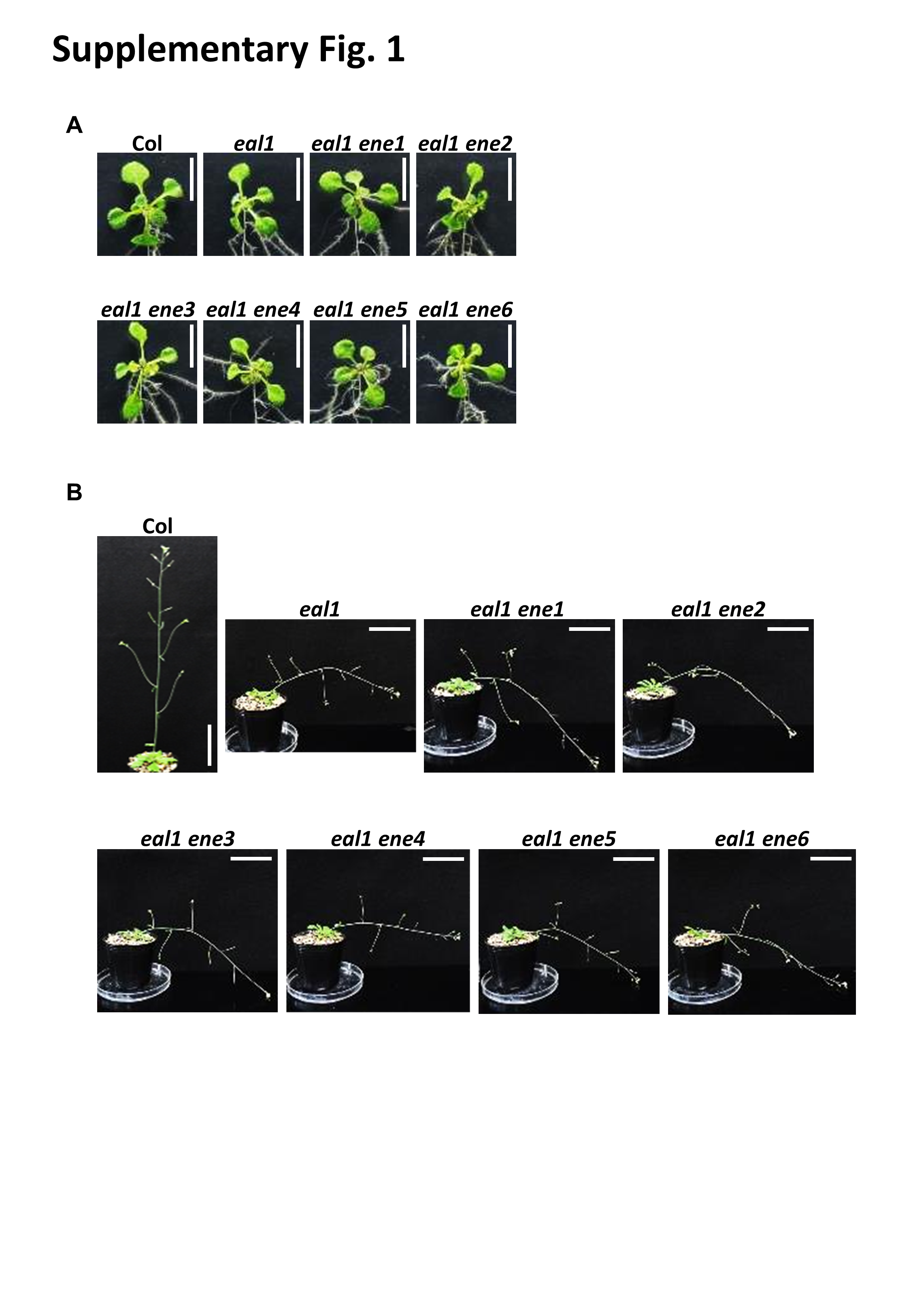

Supplement: Supplementary file 2 [file Image_1.TIF]

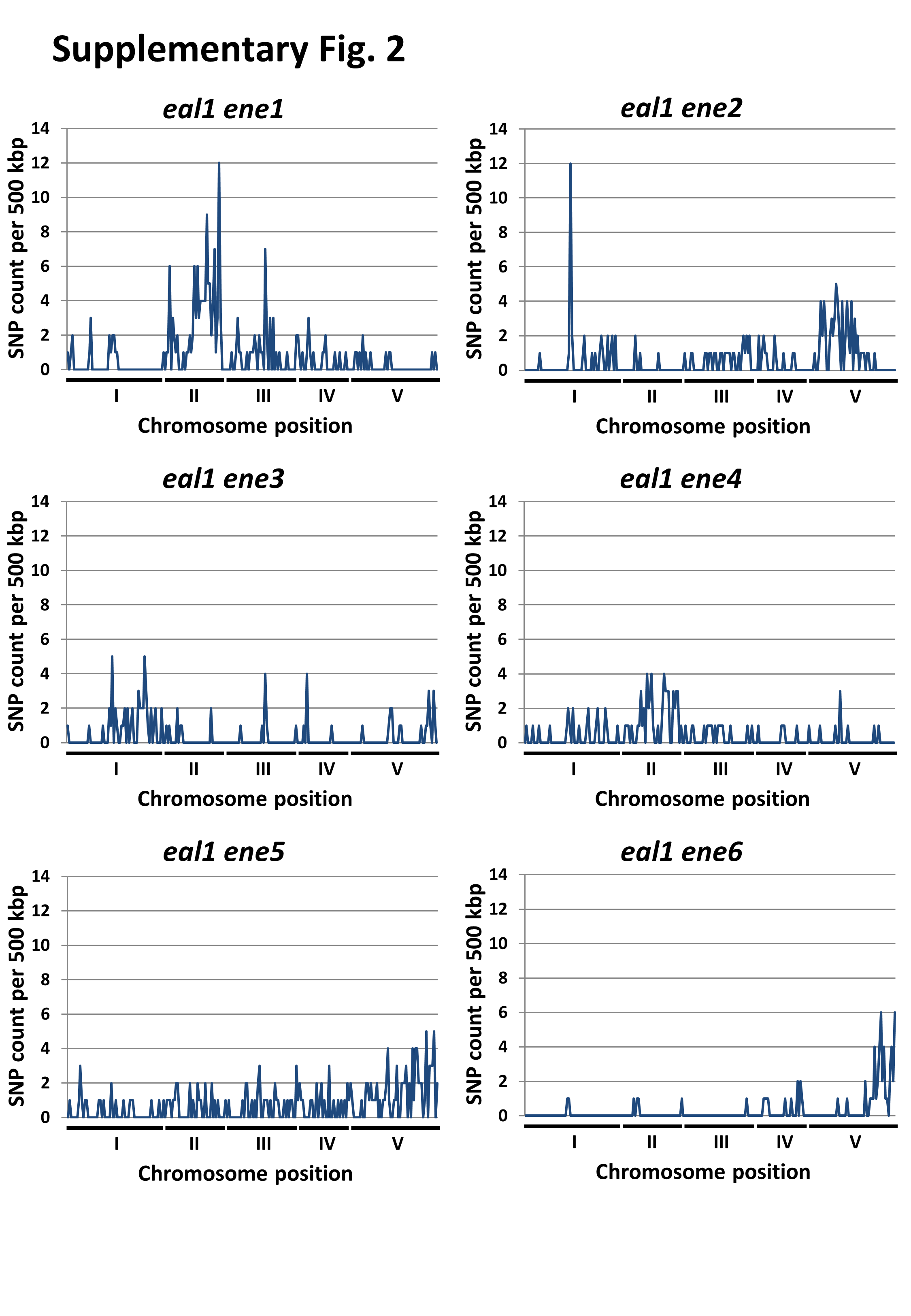

Supplement: Supplementary file 3 [file Image_2.TIF]

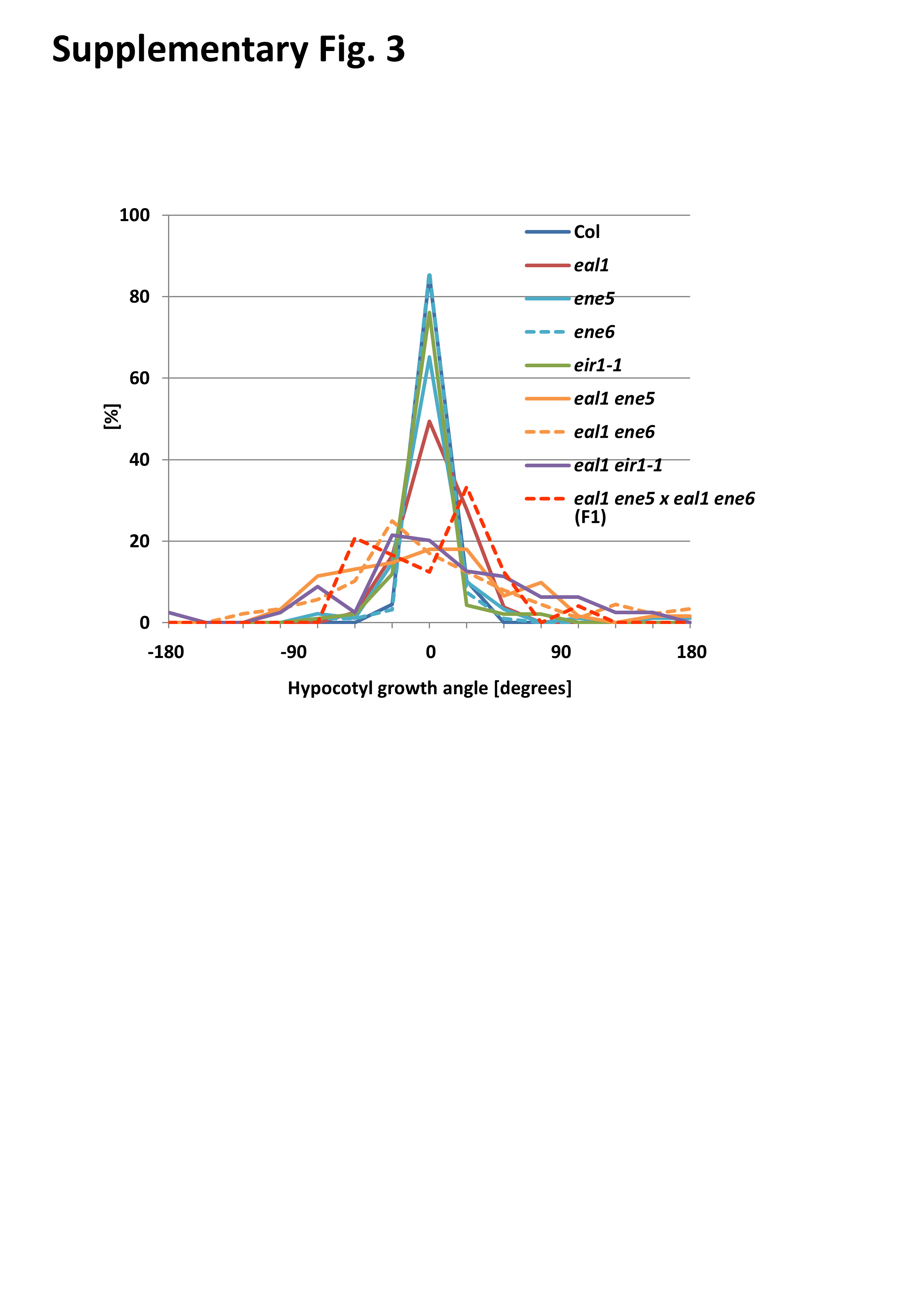

Supplement: Supplementary file 4 [file Image_3.TIF]
